# Interventional Radiology and the Care of the Oncology Patient

**DOI:** 10.1155/2011/160867

**Published:** 2011-03-29

**Authors:** Siobhan B. O'Neill, Owen J. O'Connor, Max F. Ryan, Michael M. Maher

**Affiliations:** Department of Radiology, University College Cork, Cork, Ireland

## Abstract

Interventional Radiology (IR) is occupying an increasingly prominent role in the care of patients with cancer, with involvement from initial diagnosis, right through to minimally invasive treatment of the malignancy and its complications. Adequate diagnostic samples can be obtained under image guidance by percutaneous biopsy and needle aspiration in an accurate and minimally invasive manner. IR techniques may be used to place central venous access devices with well-established safety and efficacy. Therapeutic applications of IR in the oncology patient include local tumour treatments such as transarterial chemo-embolisation and radiofrequency ablation, as well as management of complications of malignancy such as pain, organ obstruction, and venous thrombosis.

## 1. Introduction

Management of malignancy is now in the domain of the multi-disciplinary team and Interventional Radiology (IR) is occupying a prominent role in this environment [1, 2]. IR input begins with establishing the initial diagnosis of cancer, and involvement now extends to minimally invasive treatment of malignancy, often in combination with other modalities. IR has also assumed an important place in the management of the complications of malignancy, which may result from malignancy itself or secondary to treatment. This paper provides an updated overview of the role of IR in the management of the oncology patient.

## 2. Interventional Radiology in the Diagnosis of Cancer

**Figure 1:**
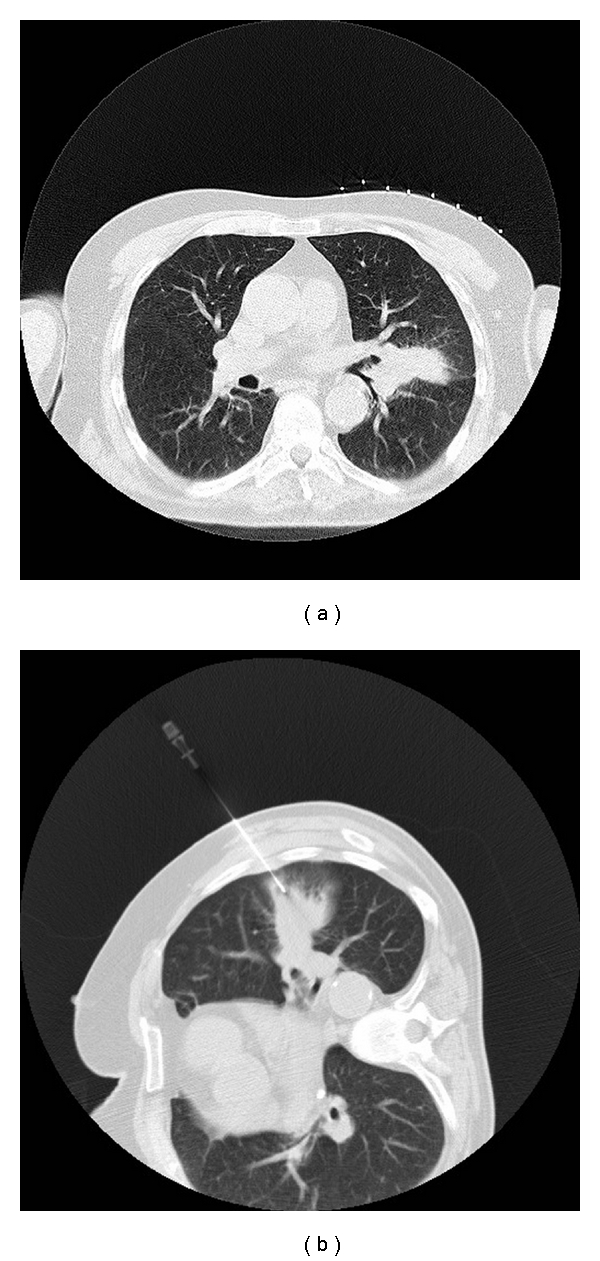
64-year-old man with lung lesion: (a) CT scan of chest in supine position shows left-sided lung lesion. (b) A 19-gauge needle has been positioned within the lesion under CT guidance and core biopsy is then performed using coaxial technique.

**Figure 2:**
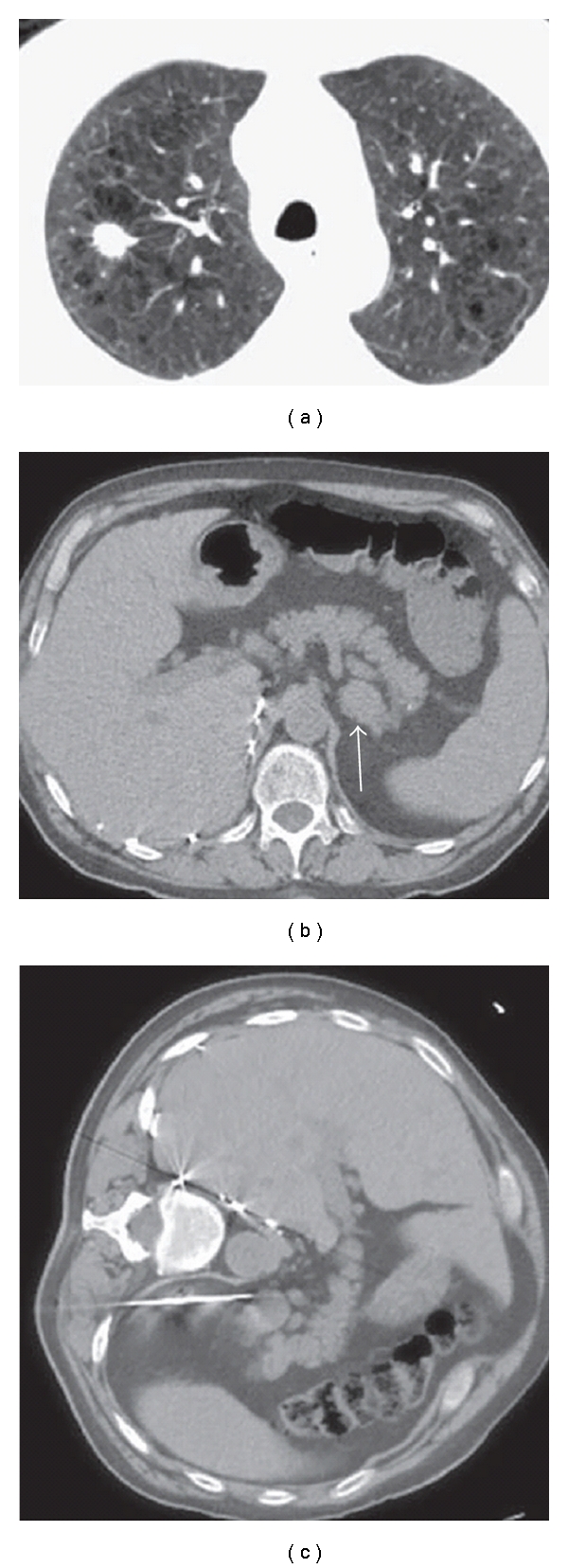
72-year-old-man with right lung mass and adrenal mass: biopsy of adrenal mass facilitates histological diagnosis and definitive staging in one procedure. (a) CT scan shows right upper lobe spiculated nodule. There is severe background emphysema, which increases the risk associated with percutaneous lung biopsy. (b) CT scan of the upper abdomen shows a left adrenal mass (arrow), suspicious for metastatic disease. (c) Percutaneous biopsy of left adrenal gland in left lateral decubitus position achieves histological diagnosis and confirms advanced staging. The risk of pneumothorax is avoided.

Appropriate treatment of malignancy is dependent on a timely definitive diagnosis and on accurate staging of disease. While non-invasive imaging techniques have improved assessment and staging for cancer, histologic confirmation remains the gold standard for definitive diagnosis of many tumours. Biopsies to establish histological diagnosis are increasingly performed using minimally invasive techniques by interventional radiologists [3]. The direct visualisation enabled by image guidance during biopsy permits safe passage of a needle into an organ or mass, improving efficacy and minimising trauma to surrounding structures (Figure 1). These minimally invasive techniques are applicable to a wide range of biopsy sites and, in most organ systems, have been demonstrated to be highly accurate with a low complication rate [3]. In biopsy planning, modern cross-sectional imaging techniques help define lesion location, accessibility, and suitability for biopsy and aid in ensuring the correct lesion is sampled in the context of multiple lesions. In selected cases where lesions are present in more than one organ, percutaneous biopsy may be used to concurrently confirm histological diagnosis and establish oncological staging by sampling the lesion suspicious for metastasis [4] (Figure 2). With improving histological and cytological techniques, particularly in immunohistochemical analysis, histological and possibly molecular examination may determine with more certainty the probable underlying primary tumour site and can predict sensitivity to chemotheraputic drugs in some cases [5]. In cases where surgical biopsy remains the preferred diagnostic approach, pre-operative tumour localisation can be performed with image guidance in many situations; an example of this is wire-localisation prior to excisional breast biopsy [6] and in the chest to guide video-assisted thorascopic surgery (VATS) for removal of lung nodules that would otherwise require open thoracotomy [7]. Increasingly, percutaneous biopsy is utilised for microbiological diagnosis of lesions suspicious for opportunistic infections (particularly fungal) in oncology patients with febrile neutropenia [8]. Choice of image guidance modality is multifactorial and there are many available options. Ultrasound offers the benefit of real-time imaging allowing accurate monitoring of the needle trajectory through tissues en route to the target lesion, with the dual advantage of avoiding patient and staff exposure to ionising radiation during the biopsy [9]. When lesions are visible by ultrasound, with suitable equipment and appropriate operator experience, this modality can provide equivalent or superior guidance to CT at time of biopsy [9]. CT guidance offers enhanced anatomical detailing and delineation with more precise needle localisation when compared to ultrasound [9]. Complications, if any, are easily recognised on CT scan. It finds particular utility in thoracic, pelvic, and retroperitoneal biopsies which are frequently difficult to perform under ultrasound guidance [8]. The main disadvantage is exposure to ionising radiation; both patient and, to a lesser extent, staff are exposed to this at time of biopsy, and the extent of such radiation exposure is related to the total scan time, scan parameters such as peak tube kilovoltage (kVp) and milliamperage (mA), the body part imaged, and the size of the patient. CT fluoroscopy is an additional tool which allows near real-time imaging of needle trajectory, which when appropriately used will shorten procedure duration [10]. Fluoroscopic images are acquired at a lower milliamperage (mA) than standard CT guidance permitting lower radiation dose to the patient, though the radiation dose to physician and assisting staff is increased [11]. Use of recently available modality fusion image guidance systems during ultrasound-guided procedures, where there is real-time projection of a needle or probe onto a pre-existing CT or MRI image, improves accuracy of needle placement while reducing radiation exposure to patient, physician, and staff [12].

## 3. Interventional Radiology in the Treatment of Cancer

### 3.1. Central Venous Access

An integral part of care of the cancer patient is intermediate and longer-term vascular access as a means of medication, chemotherapy, or parenteral nutrition administration, as well as allowing repeated blood sampling without need for venepuncture. On an annual basis in the UK, over 200,000 central access devices are inserted, many in oncology patients and while previously inserted by anaesthetists and surgeons, IR techniques are now commonly employed to site these devices [13]. Image-guided percutaneous central venous access involves placement of a catheter with its tip at the cavoatrial region or right atrium with assistance of real-time imaging, usually fluoroscopy or ultrasonography [14]. Intraprocedural complications of central access catheter insertion are typically related to injury to surrounding structures or catheter malposition, and thus occur less frequently when performed under image guidance than with blind technique guided by external landmarks [14–16]. The right internal jugular vein is the most commonly used central access route, but image guidance is particularly useful to map alternative access routes in difficult cases [17]. The longer-term complications of central access devices include thrombosis and infection and rates of these are unaffected by insertion technique. Patients with cancer have a 4- to 6- fold increased risk of thrombosis compared with the general population, a risk which is further increased by placement of a CVC [18, 19]. Clinically overt CVC-related deep venous thrombosis (DVT) in cancer patients can have an incidence as high as 28% [20, 21]. In cancer patients with CVC-related DVT, the incidence of clinically overt pulmonary embolism (PE) varies between 15% and 25% [20, 21]. The incidence of thromboembolism has been reported to increase with large multilumen catheters, those in left sided veins, and those inserted into patients with inherited and acquired prothrombotic tendencies [22]. Thrombotic prophylaxis with low dose warfarin and heparin has not been shown to reduce the incidence of thrombosis in patients with central access catheters, so anticoagulation prophylaxis is not currently recommended [23, 24]. No uniformly accepted method of therapeutic anti-coagulation or duration of treatment exists, with most patients treated with anticoagulation therapy for 6 weeks to a year, dependent on the extent of the thrombus, response to initial therapy, and whether thrombophilic factors persist [23].

### 3.2. Arterial Embolisation Techniques

Minimally invasive image-guided cancer treatments as an adjunct or alternative to surgery are increasingly being used in the management of malignancy [25, 26]. Delineation of the arterial supply of a solid tumour by contrast enhanced CT or MRI facilitates devascularisation of neoplastic tissue by transcatheter embolisation [27]. Bland mechanical occlusion can be achieved by Gelfoam (Upjohn, Kalamazoo, MI), polyvinyl alcohol, blood clots, Amplatzer occlusion devices, coils and embospheres (Biosphere medical, Rockland, MA) introduced into the tumour bed and lodged in the feeding vessel following fluoroscopic guided selective arterial catheterisation by IR [28–30]. This technique can be used alone as the primary modality of treatment, where interruption of the afferent blood supply to the tumour induces hypoxia and inhibits tumour growth, or in conjunction with ablative treatments or conventional surgery [28]. In the case of hepatic neoplasms, absence of arterial phase enhancement of a previously hypervascular lesion when reimaged 4 to 6 weeks following treatment suggests success [25, 31]. Arterial embolisation also has a role prior to surgical resection of hypervascular tumours in an effort to reduce operative blood losses. In a palliative setting, embolisation may be used to reduce tumour burden and aid symptomatic relief [32]. In acute haemorrhagic complications of malignancy, such as massive haemoptysis, haematemesis or pleural or peritoneal haemorrhage, IR embolisation of the bleeding vessel has therapeutic applications also [32]. 

**Figure 3:**
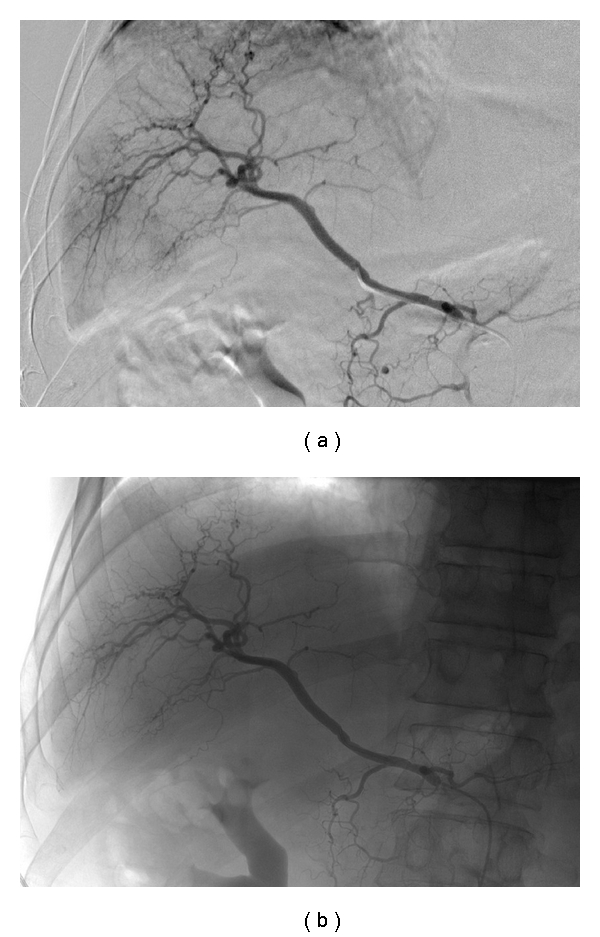
68-year-old man with hepatocellular carcinoma in segment VI of liver: (a) selective cannulation of right hepatic artery performed demonstrating tumour blush pre-embolisation with (b) absence of this tumour blush post infusion of 40 mg of doxorubicin on 300–500 *μ*m beads into this branch.

Transarterial chemoembolisation (TACE) is a modification of the above technique which is usually applied to hepatic tumours. Following selective hepatic artery catheterisation, a single or combination chemotherapy agent combined with a delivery agent, usually ethiodized oil (Ethiodol, Savage, Melville, NY), is directly infused along with an embolic agent that occludes the flow through the catheterised artery [28] (Figure 3). Hepatic tumours rely on the hepatic artery for the majority of their blood supply, as demonstrated by a tenfold greater uptake of radioisotope, in the form of radio-labelled albumen, following hepatic artery infusion when compared to portal vein infusion [33]. The advantage of TACE over systemic chemotherapy is that delivery of the chemotherapeutic agent is targeted at the lesion allowing a higher local concentration of the agent and lower systemic doses. Embolisation of the artery increases the dwell time of the chemotherapeutic agent. Chemoembolisation has the advantage of being repeatable and treatment may involve a number of sessions until the entire tumour bed is devascularised [8]. The liver tolerates this procedure because of its dual blood supply and, to avoid hepatic necrosis, chemoembolisation should be performed with caution in the absence of portal vein patency or a sufficient alternative blood supply to the liver [34]. Drug-eluting beads (DEB) comprise particles of variable size which bind and elute doxorubicin in a predictable manner. They may be used in the place of standard chemotherapeutic agent infusion during the TACE procedure in patients with hepatocellular carcinoma and have shown promising results to date, making them likely to be of therapeutic benefit in the future [35]. 

Radioembolisation, a novel form of liver-directed brachytherapy, is another modality with potential for focussed treatment of hepatic malignant lesions [36, 37]. Selective catheter placement allows introduction of beta-radiation emitting radioisotopes directly into the tumour mass by means of microspheres (glass, albumen, or resin) [38]. Depending on the nature of the tumour, various radionuclides are used, including Yttrium 90, rhenium, and holmium [36, 39]. Beta radiation has a very low penetration (approximately 2.5 mm in human tissues), thus its necrosing effects are localised [40]. The concurrent emission of a small amount of gamma radiation, which is capable of penetrating the body tissues, allows detection of the radiolabelled particles by a gamma camera and appropriate localisation of isotope can be confirmed. Accurate transcatheter delivery of radioisotopes has been shown to be safe, with efficacy confirmed on preliminary results [41, 42]. Radioembolisation has been reported to produce a meaningful response and disease stabilisation in patients with advanced unresectable liver metastases, and may be potentially very useful in patients with chemorefractory metastatic colorectal cancer [43]. Technically radioembolisation is more difficult than chemoembolisation, with potential for inadvertent nontarget embolisation of other organs, particularly the stomach, small bowel, and gall-bladder, causing slow-healing gastrointestinal ulcers or cholecystitis respectively [40]. Other potential adverse effects include pneumonitis and radioembolisation-induced liver disease, therefore use should be restricted to patients with a serum bilirubin of less than 2 mg/dL and to patients without significant hepatopulmonary shunting [35].

### 3.3. Gene Therapy

Advances in molecular oncology and tumour immunology have facilitated the development of gene therapy in the treatment of malignancy [44]. Strategies employed include stimulation of the immune response to the tumour, reduction of oncogenic expression, restoration of tumour suppressor gene function, alteration of susceptibility of proliferating tumour cells to chemotherapeutics, and modulation of angiogenesis [44, 45]. In an IR technique similar to that used in chemoembolisation, genetic agents may be administered directly into the tumour mass by selective arterial injection, after which the vessel is embolised thus limiting adverse effects and prolonging agent dwell time which is believed to improve genetic transfer rate [46]. As DNA has a limited ability to cross cell membranes, vector agents are used to optimise transfection rates and achieve adequate expression of the therapeutic molecule within a cell [46]. Common vectors include plasmids and phospholipid agents, which have short-lived effects, and viruses (retroviruses, adenoviruses, EBV), which have demonstrated more lasting genetic expression. Clinical experience with these therapies to date is limited with studies limited to small patient cohorts who have failed conventional therapies. However, results in treatment of hepatic neoplasms and their metastases appear promising [47, 48].

### 3.4. Ablative Techniques

Local tumour ablation is an alternative method of achieving tumour control in those patients with early stage malignant disease, particularly in the liver, who are not candidates for resection. IR mediated tumour ablation induces tumour necrosis by the application of energy and modalities employed include radiofrequency (RF), laser, microwave, ultrasound and cryotherapy [49]. Radiofrequency ablation (RFA) involves administration of electromagnetic energy in the radiofrequency range to a tumour by means of a locally placed electrode connected in a closed loop circuit to a monopolar or bipolar energy source [50]. Tissues immediately surrounding the electrode tip are heated to temperatures in excess of 60 degrees Celsius with consequent thermal damage to the surrounding tissues and cell death [50]. RFA has been demonstrated to be safe with a mortality rate of 0.3% and a major complication rate of 2.2% [51], and has gained acceptance as a method of managing hepatic and lung malignant disease, with efficacy also described in the treatment of adrenal, renal, and skeletal lesions [25, 52, 53]. While RFA is the most commonly used ablative means, other modalities are finding increasing clinical use. Cryoablation results in cell death through the application of subfreezing temperatures, achieved by use of argon gas under high pressure [54]. Alternating cycles of freezing and thawing results in cell death due to the associated mechanical stresses upon cell membranes with phase change and ice formation and microvascular thrombosis induces tissue ischaemia which limits bleeding [55]. The application of electromagnetic energy in the microwave range (at least 900 MHz) agitates water molecules in targeted tissues, resulting in frictional heat and cell death via coagulation necrosis [56]. Though direct comparison of modalities is difficult, as a therapeutic tool, microwave ablation has been shown comparable in efficacy to RFA, particularly for the treatment of hepatocellular carcinoma [57], however, RFA achieves a lower local recurrence rate, higher survival rate, and extensive necrosis after only a few treatment sessions [58]. Potential added benefits of microwave ablation over RFA include larger tumour ablation volumes, optimal heating of cystic masses, and less procedural pain [59]. The involutional changes that occur following necrosis should be monitored by serial imaging following ablation, with specific postablation CT and MR imaging protocols being developed at many institutions in an effort to confirm completeness of ablation and to detect residual or recurrent disease [8, 60].

## 4. Interventional Radiology in the Management of the Complications of Cancer

Malignancy can induce dysfunction of many organs and bodily systems. Though debilitating, a significant portion of these complications are reversible, many by minimally invasive IR methods. Such treatment can relieve symptoms, alleviate pain, and improve operability of patients, thus having a significant positive impact on quality of life. 

### 4.1. Biliary Obstruction

The majority of patients presenting with malignant biliary obstruction have an underlying pancreatic neoplasm extrinsically compressing the distal bile duct and can be treated by endoscopic means [61]. Metastatic disease at the hepatic hilar nodes or in the peripancreatic nodes may cause obstructive jaundice from extrinsic pressure on the proximal portions of the biliary tree and may require percutaneous intervention if less invasive endoscopic means fails to achieve adequate biliary decompression. Contrast injection into an intra-hepatic bile duct at percutaneous transhepatic cholangiography will delineate the anatomy of the biliary tree, determining the location of obstruction, and helping to guide intervention [62]. Percutaneous transhepatic biliary drainage (PBD) is an effective method for the primary or palliative management of many biliary abnormalities demonstrated with cholangiography. This procedure involves selective cannulation of the biliary tree with catheter manipulation, then placement of a catheter or stent to facilitate internal or external drainage of biliary flow and so allow decompression of the biliary system [8]. Metal stents have a six-month patency rate of 50% and are thus almost exclusively used for malignant disease [63, 64]. The percutaneous treatment of biliary lesions is frequently staged, requiring several sessions to achieve therapeutic goals, though, in the majority of patients, liver function indices improve following a single treatment [62]. PBD can be associated with major complications including sepsis, haemorrhage and localised infective and inflammatory processes (abscess, peritonitis, cholecystitis, and pancreatitis) [64]. The incidence of complications is higher in the oncology than in the general population, perhaps related to advanced malignancy and the potential presence of coexisting immunosuppression [64, 65]. The incidence of cholangitis in oncology patients undergoing PBD approaches 50%, such infection observed twice as commonly in those with internal and external drainage than in those with external drainage alone [66]. The longer the duration of PBD is the more likely the patient is to develop cholangitis [67]. The incidence of infected bile in patients with malignant biliary obstruction is 25% to 36% [68]. Prior to initiating percutaneous biliary procedures, all patients should be administered appropriate prophylactic antibiotics to minimise septic complications, including cover for escherichia coli, klebsiella, enterococcus, streptococcus, enterobacter and pseudomonas aeruginosa [8].

### 4.2. Renal Obstruction

**Figure 4:**
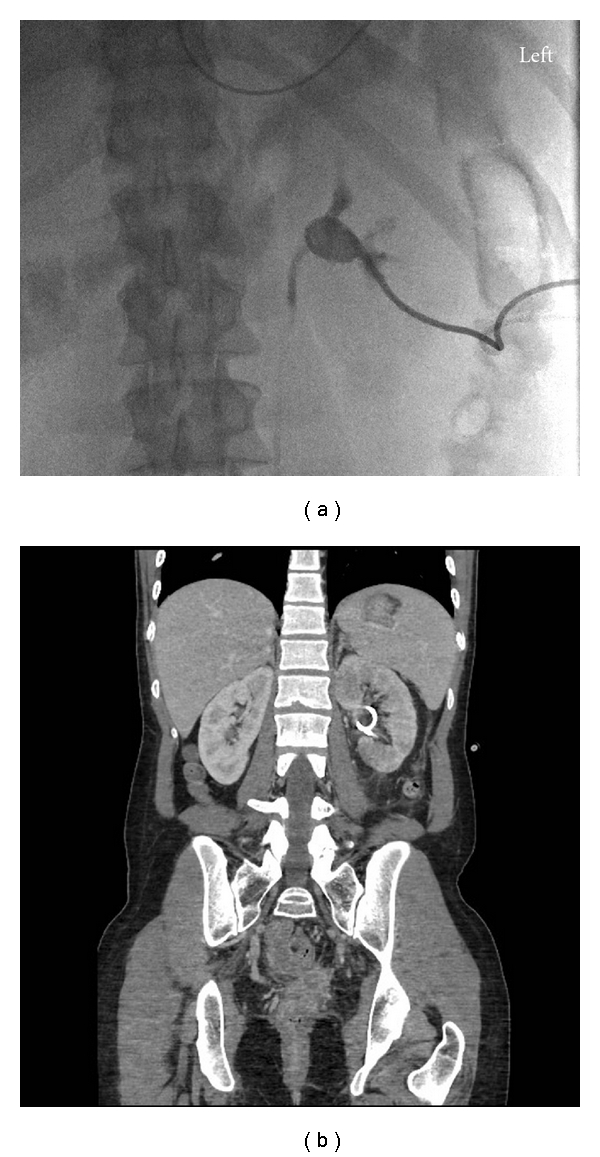
21-year-old woman with cervical cancer develops ureteric obstruction due to local disease invasion. (a) Contrast injection immediately following percutaneous nephrostomy confirms 8 french catheter in good position within renal collecting system. (b) Contrast-enhanced CT scan confirms percutaneous nephrostomy catheter in positioned within left renal collecting system. Patient is post total abdominal hysterectomy and bilateral salpingo-oophorectomy.

Malignant ureteral obstruction is an ominous sign in the cancer patient and may be due to extrinsic tumor compression, retroperitoneal adenopathy, or direct tumor invasion [69]. Ureteral obstruction can be induced by a wide range of malignancies, most commonly those of gastrointestinal, urologic, or gynaecologic origin, and may be unilateral or bilateral. Management requires urinary decompression, often by means of percutaneous nephrostomy (PCN). PCN is the most common renal intervention performed by IR and, by providing direct access to the urinary tract, allows drainage of tract contents as well as providing access for further uroradiologic intervention via the route established [70]. Indications for PCN in the emergent setting include urinary tract sepsis, pyonephrosis, deteriorating renal function, or electrolyte disturbances such as hyperkalemia and metabolic acidosis [8]. Potential benefits of urinary decompression and diversion by this means include reduction in the incidence of gram-negative septicaemia due to renal obstruction, partial recovery of renal function, reversal of metabolic disturbance, and reduced inpatient admission times. Image guidance may be provided with fluoroscopy, ultrasound, or often a combination of both modalities [71] (Figure 4). The size and type of the drainage catheter should be chosen appropriately according to the nature of the fluid to be drained [70]. In cases of malignant ureteric obstruction, when retrograde stenting is unsuccessful or not feasible, percutaneous dilatation of the stricture may be achieved antegradely through the PCN tract where, under fluoroscopic guidance, a catheter is manipulated across the stenotic region and the lesion is progressively dilated by catheter advancement, ureteral dilator, or by inflating balloons of appropriate diameter and length [70]. After dilatation, an internal ureteral stent, or internal-external nephroureteral catheter is placed to prevent restenosis [70, 72]. Plastic stents are favoured over metal ones because they induce less urothelial hyperplasia and can be easily replaced. In a series of 102 cases of malignant ureteral obstruction (68% bilateral), initial management with PCN or ureteral stent achieved successful decompression of the system in 95% of cases [73]. The rate of successful completion of PCN in oncology is mainly determined by the degree of dilatation of the collecting system and by the patient's body habitus [74]. However, in the above series, significant complications such as infection and catheter blockage were observed in 53% of patients and overall survival was poor with a median of seven months [73]; these outcomes may appear on initial reflection to be disappointing, but are most likely explained by advanced stage of malignancy in most patients.

### 4.3. Upper Gastrointestinal Obstruction

**Figure 5:**
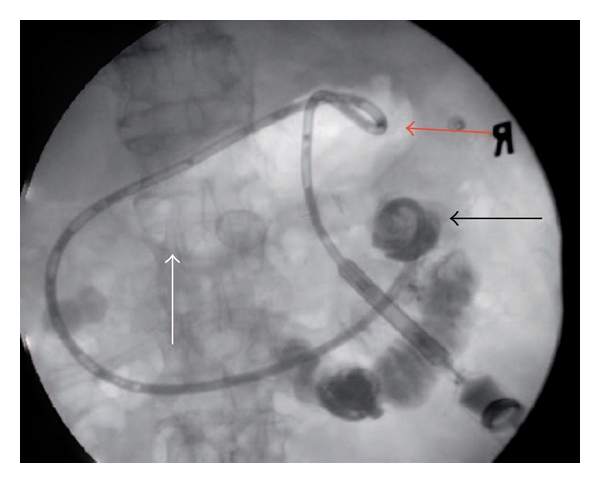
73-year-old man with pancreatic cancer: percutaneous gastrojejunostomy catheter placed for feeding. Contrast injection following placement of percutaneous gastrojejunostomy tube confirms that the tip of the tube is in excellent position in the jejunum (arrow). Note the gas-filled stomach (white arrow) and the locking pigtail catheter in stomach which serves to maintain catheter in position and prevent dislodgement.

Patients with head, neck, or oesophageal malignant lesions are, due to luminal obstruction or swallow impairment, frequently unable to tolerate adequate oral intake and require nutritional support, often by gastrostomy or gastrojejunostomy [75]. The interventional radiologist can play an important role in the provision of enteral alimentation to these patients (Figure 5). Percutaneous image-guided placement of feeding tubes has demonstrated higher technical success rates and is considered safer than endoscopic or surgical placement [76]. In addition, it may be successfully performed in patients in whom conventional endoscopy is impossible [75]. Among the more common early complications of gastrostomy insertion are infection and mild discomfort on feeding, which have been observed in 23% and 33% of cases respectively [77, 78]. Tube dislodgement is relatively common; however, if the tract is established for more than two weeks, it is frequently possible to access the tract and reinsert the tube without the need for repuncture of the stomach [8]. Complication rates are similar with gastrostomy and gastrojejunostomy. Malignant small bowel obstruction, as seen in patients with peritoneal carcinomatosis, often of ovarian origin, is a further indication for gastrostomy or gastrojejunostomy as a means of decompression, with a technical success rate in the region of 98% [79]. The presence of ascites in such patients mandates paracentesis prior to procedure as peritoneal fluid leads to technical difficulty and the risk of pericatheter leakage and the possibility of peritonitis [80–82]. Gastropexy is advised prior to gastrostomy to reduce the likelihood of catheter dislodgement from the anterior abdominal wall and to reduce risk of peritonitis and peri-catheter leakage [8, 80, 82].

### 4.4. Pleural Space Intervention

Malignant pleural effusions, often related to pleural and lymphatic involvement, are a significant source of morbidity in the oncology patient, presenting with dyspnoea, cough, and chest pain [83]. As a malignant pleural effusion is a preterminal event with a mean survival of three months, the usual aim of treatment is palliation, and relief of symptoms and prevention of recollection [84, 85]. Successful drainage can be achieved by IR with catheter placement under fluoroscopic, ultrasound or CT guidance. Image-guided needle aspiration of pleural fluid collections may also be performed to evaluate for the presence of malignant cells using cytology, thus aiding in the initial diagnosis of malignancy or staging of known disease [85]. 

Therapeutic thoracocentesis provides temporary symptomatic relief until the effusion reaccumulates, as is often the case in the setting of malignant effusions, necessitating a repeat procedure. Definitive treatment requires pleurodesis [86]. Prevention of recurrent pleural effusions can be achieved by chemical or talc pleurodesis. Prior to pleurodesis, large effusions require drainage to optimise success rates of pleurodesis and to prevent accumulation of therapeutic agents within the pleural space. Based on efficacy and the likelihood of recurrence, thoracoscopic pleurodesis is the preferred technique but has the drawback of requirement for a general anaesthetic [86]. IR pleurodesis, entailing instillation of the sclerosing agent via a thoracostomy tube once complete evacuation of the effusion has occurred, can be performed at the bedside and is generally well tolerated [8]. Available evidence supports the need for chemical sclerosants to achieve successful pleurodesis, with talc as the agent of choice [86]. Other agents employed include tetracycline, bleomycin, and mustine.

### 4.5. Pain

A significant source of cancer-related morbidity, particularly in advanced disease, is pain. Prevalence can range from 40% to as high as 90% with advanced disease [87, 88], and when inadequately controlled, the impact of pain can be profound. Opiates, with their considerable side effect profile, remain the mainstay of treatment and pain can be well managed in 80% to 90% of cancer patients according to the principles of the World Health Organisation (WHO) analgesic ladder [89–91]. Patients who have pain that is not controlled by these means, or who have well-controlled pain but with intolerable analgesic side effects, may benefit from interventional pain management measures. As techniques expand, IR is assuming an evolving role in the management of cancer-associated pain. However, while IR has a role in the treatment of oncological pain, it is noteworthy that IR interventions may themselves be a source of significant pain and discomfort among patients, particularly procedures involving drainage of the renal and biliary tracts [92]. Optimal analgesia during and after such procedures is essential. Percutaneous vertebroplasty, in recent years, has emerged as an effective minimally invasive treatment for severe and refractory pain secondary to vertebral fracture [93, 94]. In particular, its use has been met with considerable success in the treatment of painful osteoporotic vertebral compression fractures, where fracture stability is achieved by introduction of cement. It has also found less frequent use in the treatment of fractures secondary to neoplastic disease [94, 95]. Osteolytic processes, such as myeloma, often induce fractures, resulting in instability and pain. Vertebroplasty has been shown to reduce requirements for analgesia and is now being utilised in the treatment of vertebral fractures which result from malignant osseous infiltration [95]. Again, the complication risk is higher among oncology patients than in the general population, with rates of 5% and 1%, respectively for major complications [95]. The more significant complications include leakage of cement into the spinal canal, pulmonary embolus, and pulmonary oedema. The beneficial effects of pain reduction and improved mobility are observed in 50%–60% of oncology patients undergoing vertebroplasty, with better results achieved by treating subacute rather than chronic fractures [96]. 

Neuropathic pain associated with upper abdominal visceral tumours is frequently poorly responsive to analgesic therapy [97]. When resistant to analgesics, celiac ganglion neurolysis and nerve block can achieve successful palliation of pain in the majority of patients, particularly that related to pancreatic, gastric, oesophageal, and biliary malignancies [97]. Agents employed include local alcohol and phenol, which induce permanent nerve root destruction, and triamcinlone, which causes reversible nocireceptor blockade [98]. A variety of imaging modalities can be used to guide celiac axis block; CT is most commonly used with either an anterior or posterior approach, dependent on operator experience and anatomic considerations in the individual patient [99]. Reported minor complications include transient diarrhoea in 73% and orthostatic hypotension in 12% [100].

### 4.6. Venous Thromboembolic Disease

Malignancy is an established risk factor for venous thromboembolism. Fifteen percent of cancer patients develop a symptomatic venous thrombosis in the course of their therapy, and 50% have evidence of venous thrombosis at autopsy [101]. Vena caval filters, intravascular devices designed to prevent pulmonary embolus by trapping venous emboli, are an accepted method of managing venous thromboembolism in the oncology patient. Indications for insertion include the occurrence of a lower limb deep venous thrombosis in patients for whom anticoagulation is contraindicated, in those in whom a complication of anticoagulation has occurred, or in those who develop recurrent PEs despite adequate anticoagulation [102]. In experienced hands, the technical success rate of inferior vena caval filter placement is over 97% [102]. Empirical use is not at present supported in the literature. 

Recent developments in endovascular technologies have provided radiologists with an assortment of minimally invasive, catheter-based strategies to manage venous thrombus, including both deep venous thromboses and pulmonary emoboli. These percutaneous treatment methods for venous thrombotic conditions include catheter-directed thrombolysis, percutaneous mechanical thrombectomy devices, and adjuvant venous angioplasty and stenting. Catheter-directed thrombolysis therapy involves the use of infusion catheters and wires to achieve local high-dose delivery of thrombolytic agents to the thrombus with the aim of achieving more rapid lysis. This allows a more predictable thrombolytic effect with a lower risk of haemorrhagic complications and with higher patency rates than systemic thrombolysis, with the added benefit of ability to visualise the entire venous system prior to and after administration of the pharmacologic agent [103]. Percutaneous mechanical thrombectomy may be used as a primary therapy for an acute thrombotic event, for thrombus involving large vessels such as the vena cava, or, more commonly, for patients in whom, despite conventional anti-coagulation or catheter-directed therapy, there is persistent thrombus. Such therapy also has a role in patients with contraindications to continuous anti-coagulation or the use of thrombolytic agents. It is however suggested that, in the absence of contraindications to the use of thrombolytic agents, mechanical thrombectomy devices should be used in conjunction with pharmacological thrombolysis [103]. Mechanical, chemical, and hybrid pharmacomechanical thrombectomy devices are available for clot extraction with variable success rates [104], though further discussion of these devices is outside the scope of this paper.

## 5. Conclusion

With the expanding application of minimally invasive techniques to the investigation and management of malignancies, the interventional radiologist is assuming a more prominent role in the multidisciplinary team that cares for the patient with cancer. The use of IR techniques in oncology patients should be evidence based to ensure optimal outcome and minimise potential complications.
